# ING5 knockdown enhances migration and invasion of lung cancer cells by inducing EMT via EGFR/PI3K/Akt and IL-6/STAT3 signaling pathways

**DOI:** 10.18632/oncotarget.17346

**Published:** 2017-04-21

**Authors:** Xin-Li Liu, Xu-Tao Zhang, Jin Meng, Hong-Fei Zhang, Yong Zhao, Chen Li, Yang Sun, Qi-Bing Mei, Feng Zhang, Tao Zhang

**Affiliations:** ^1^ Key Laboratory of Gastrointestinal Pharmacology of Chinese Materia Medica of the State Administration of Traditional Chinese Medicine, Department of Pharmacology, School of Pharmacy, Fourth Military Medical University, Xi’an, China; ^2^ Department of Thoracic Surgery, Tangdu Hospital, Fourth Military Medical University, Xi’an, China; ^3^ Department of Pharmcy, Hospital of PLA, Beijing, China; ^4^ Laboratory Animal Center, Fourth Military Medical University, Xi’an, China

**Keywords:** ING5, lung cancer, epithelial mesenchymal transition (EMT), EGFR/PI3K/Akt, IL-6/STAT3

## Abstract

ING5 belongs to the Inhibitor of Growth (ING) candidate tumor suppressor family, whose functions have been involved in the regulation of chromatin remodeling, cell cycle progression, proliferation and apoptosis. Our previous study has shown that ING5 overexpression inhibits lung cancer aggressiveness via suppressing epithelial to mesenchymal transition (EMT). However, the mechanisms remain largely unknown. In the current study, by Phospho-Kinase array and western blot, we have defined significantly upregulated EGFR/PI3K/Akt and IL-6/STAT3 oncogenic signaling pathways in ING5 knockdown A549 cells, which could be downregulated by ING5 overexpression. PI3K inhibitor ZSTK474 or STAT3 inhibitor Niclosamide not only abolished ING5 knockdown-promoted proliferation, colony formation, migration and invasion of lung cancer A549 cells, but also impaired ING5 knockdown-stimulated metastasis of cancer cells in mouse xenograft models with tail vein injection of A549 cells. Furthermore, treatment with ZSTK474 or Niclosamide decreased protein level of EGFR, p-Akt, IL-6 and p-STAT3, and reversed ING5 knockdown-promoted EMT, as indicated by downregulated expression of EMT marker E-cadherin, an epithelial marker, increased expression of N-cadherin, a mesenchymal marker, and EMT-related transcription factors including Snail, Slug, Smad3 and Twist. Taken together, these results demonstrate that loss of ING5 enhances aggressiveness of lung cancer cells by promoting EMT via activation of EGFR/PI3K/Akt and IL-6/STAT3 signaling pathways.

## INTRODUCTION

The proteins of the Inhibitor of Growth (ING) candidate tumor suppressor family include ING1-ING5, which share a highly conserved carboxy-terminal plant homeodomain (PHD) and are involved in multiple cellular functions such as cell cycle regulation, senescence, apoptosis, chromatin remodeling and regulation of autophagy and differentiation [1–4]. ING5 has been identified to physically interact with p300 and p53, and overexpression of ING5 induces apoptosis in colorectal cancer cells [5]. Further study has revealed that ING5 associates with HBO1 and MOZ/MORF to form two distinct HAT complexes, and are involved in chromatin remodeling and DNA replication [6]. Our previous study [7] screened ING5 as an interaction partner of INCA1, whose overexpression in MEF cells inhibited cell growth, induced a delay in S-phase progression and increased Fas-induced apoptosis in an INCA1-dependent manner [8]. ING5 has also been identified as a component of genetic interacting network to control epidermal differentiation and protect epidermal stem cells from premature differentiation [9]. These results suggest that ING5 may function as a tumor suppressor through multiple mechanisms. Recently, we and others [10, 11] have reported that ING5 inhibits lung cancer migration and invasion by preventing EMT, proposing an anti-metastasis role of ING5. However, the underlying molecular mechanisms and signaling pathways of ING5 function are still unclear.

Lung cance is the leading cause of cancer-related mortality worldwide with frequently occurred early metastasis being the principal cause. Thus, finding effective anti-metastasis therapeutic target may lead to improvement of patient outcome. The metastatic cascade represents a multi-step process, in which EMT is a crucial event in the early stage of cancer metastasis [12]. EMT is a process by which epithelial cells lose their cell polarity and cell-cell adhesion to become mesenchymal cells and gain migratory and invasive properties, and is characterised by a loss of intercellular adhesion, down-regulation of epithelial markers and up-regulation of mesenchymal markers [13, 14].

The invasive abilities of lung cancer cells are regulated by different signaling pathways, among which PI3K/AKT and STAT3 pathways are frequently activated in cancer cells resulting in tumourigenesis and progression [15–18]. Both PI3K/Akt and STAT3 pathways are also involved in different factors-induced EMT [19]. The EMT-related transcription factor Snail has been widely investigated as a downstream molecule of both PI3K/Akt and IL-6/STAT3 pathways [20–22]. We hypothesized that there could be a correlation between ING5 tumor suppressive function and PI3K/Akt and STAT3 signaling in lung cancer.

In the current study, we demonstrate, for the first time, that ING5 knockdown promotes invasion of lung cancer cells by inducing EMT via EGFR/PI3K/Akt and IL-6/STAT3 signaling pathways. PI3K/Akt inhibitor ZSTK474 or STAT3 inhibitor Niclosamide could reverse EMT and invasiveness promoted by ING5 knockdown. Our data further establish the role of ING5 as a tumor suppressor in lung cancer progression and metastasis by targeting two oncogenic signaling pathways.

## RESULTS

### Loss of ING5 promotes lung cancer invasiveness and EMT

**Figure 1:**
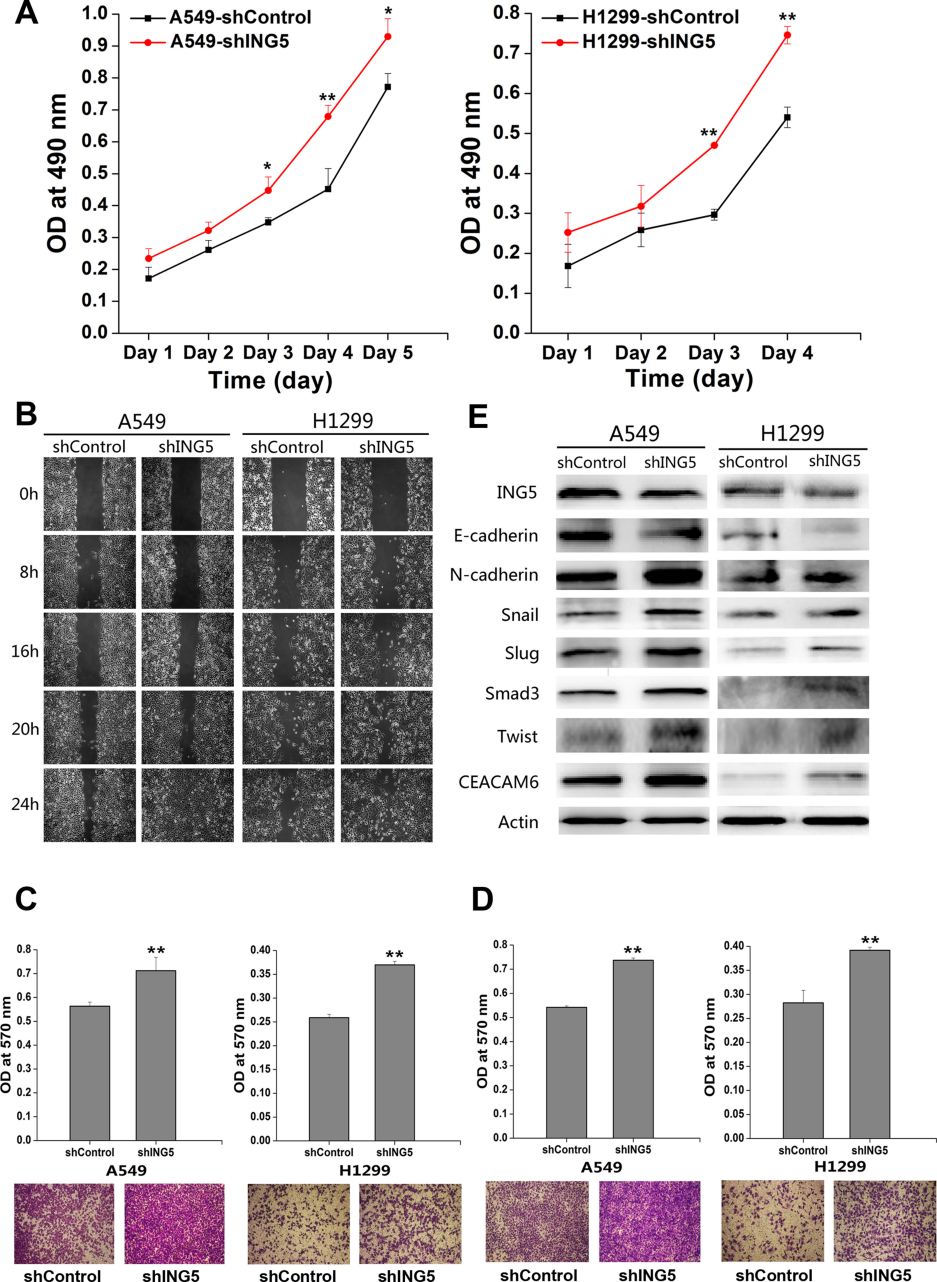
ING5 knockdown promotes lung cancer invasiveness by inducing EMT (**A**) Effects of ING5 knockdown on the proliferation of A549 and H1299 cells. Data are shown as mean plus standard error of three independent experiments. *P < 0.05 and **P < 0.01 compared to corresponding control. (**B**) Effects of ING5 knockdown on migration of A549 and H1299 cells by wound-healing assay. A scratch wound was made on cell surface and cells were photographed at 0 h, 8 h, 16 h, 20 h and 24 h. Representative pictures are shown. (**C**) Effects of ING5 knockdown on migration of A549 and H1299 cells. The migrated cells were photographed (100 × magnification). Representative pictures are shown. The migrated cells were quantified by the absorbance of the crystal violet washed with 33% acetic acid. Data are shown as mean plus standard error of three independent experiments. **P < 0.01 compared to corresponding control. (**D**) Effects of ING5 knockdown on invasion of A549 and H1299 cells. The invaded cells were photographed (100 × magnification). Representative pictures are shown. The invaded cells were quantified by the absorbance of the crystal violet washed with 33% acetic acid from the cells that invaded the underside of the porous polycarbonate membrane. Data are shown as mean plus standard error of three independent experiments. **P < 0.01 compared to corresponding control. (**E**) Effects of ING5 knockdown on protein expression of EMT markers and related proteins by western blot. Actin was used as an internal loading control.

We used ING5 overexpression and knockdown cells lines described previously [10] to further confirm ING5 function in cancer invasiveness. The effects of ING5 knockdown on cell proliferation was observed by proliferation assay. ING5 knockdown significantly increased proliferation of A549 and H1299 cells from 48 h after cell adhesion (Figure 1A). Knockdown of ING5 promoted migration of lung cancer A549 and H1299 cells as assessed by wound-healing assay and transwell migration assay (Figure 1B, 1C). ING5 knockdown also significantly accelerated lung cancer cells to invade through Matrigel-coated polycarbonate filter in the transwell chamber (Figure 1D). Previously, we have demonstrated that ING5 overexpression increased E-cadherin, while decreased N-cadherin, Snail and Slug, which could be reversed by ING5 knockdown in A549 cells. The current study further confirmed the results in H1299 cells with ING5 overexpression and knockdown (Figure 1E, Supplementary Figure 1). We also defined the EMT-inducing transcription factors Smad3 and Twist, and EMT-inducing protein CEACAM6 which could be down regulated by ING5 overexpression and increased by ING5 knockdown in both A549 and H1299 cell lines (Figure 1E, Supplementary Figure 1). Taken together, these results indicate that loss of ING5 promotes lung cancer invasion by inducing EMT.

### ING5 knockdown activates EGFR/PI3K/Akt and IL-6/STAT3 signaling pathways

**Figure 2:**
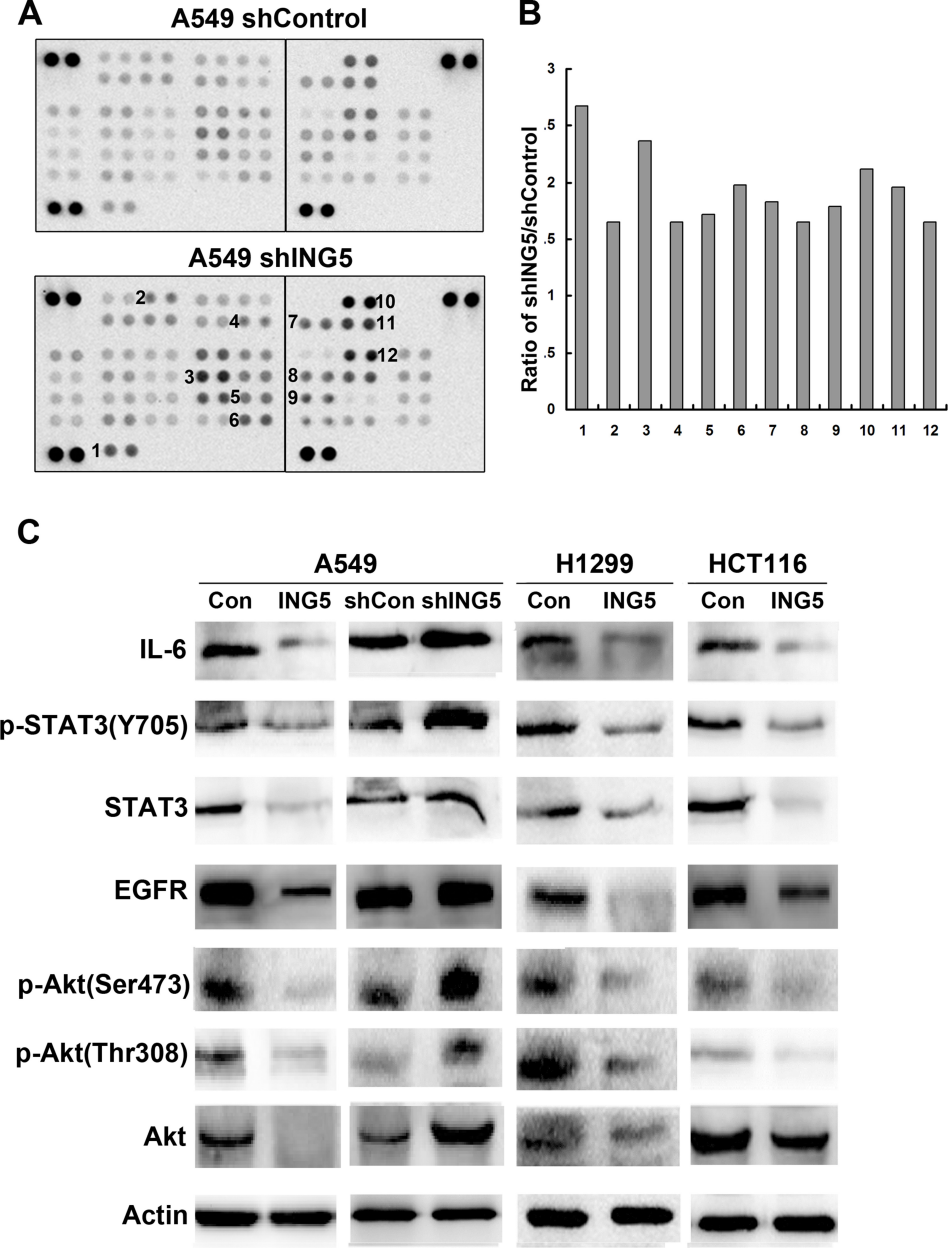
ING5 knockdown activates EGFR/PI3K/Akt and IL-6/STAT3 signaling pathways (**A**) The Phospho-Kinase antibody array was incubated with cell lysates from shControl and shING5 A549 cells. Data shown are from a 2 minute exposures to film. (**B**) Twelve pairs of duplicate spots representing 12 proteins were selected and the average signal density was calculated. The relative change of protein phosphorylation upon ING5 knockdown was determined by the ratio of corresponding signals from shING5 to shControl array film. 1. PRAS40 (T246); 2. ERK1/2 (T202/Y204, T185/Y187); 3. STAT2 (Y689); 4. Akt1/2/3 (S473); 5. STAT5b (Y699); 6. STAT5a/b (Y694/Y699); 7. Akt1/2/3 (T308); 8. P70S6 Kinase (T421/S424); 9. STAT3 (Y705); 10. P53 (S392); 11. P53 (S46); 12. P53 (S15). (**C**) Effects of ING5 overexpression or knockdown on protein expression involved in PI3K and STAT3 pathways. Protein level of total Akt, P-Akt S473, P-Akt T308, total STAT3, P-STAT3 Y705, EGFR and IL-6 were detected by western blot. Actin was used as an internal loading control.

To analyze the signaling pathways and mechanisms of ING5 knockdown-induced cancer invasiveness, we did antibody array with 43 kinase phosphorylation sites (Figure 2A, Supplementary Figure 2). Fold change of density of each target was calculated and listed in Supplementary Table 1. We chose 12 targets which showed higher phosphorylation level in ING5 knockdown A549 cells (Figure 2B), including 1. PRAS40 (T246); 2. ERK1/2 (T202/Y204, T185/Y187); 3. STAT2 (Y689); 4. Akt1/2/3 (S473); 5. STAT5b (Y699); 6. STAT5a/b (Y694/Y699); 7. Akt1/2/3 (T308); 8. P70S6 Kinase (T421/S424); 9. STAT3 (Y705); 10. P53 (S392); 11. P53 (S46); 12. P53 (S15). Among these proteins, PRAS and P70S6 kinase are downstream targets of PI3K/Akt signaling. We then focused on PI3K/Akt and STAT3 signaling pathways, both of which have been extensively studied to promote cancer cell proliferation and invasion, to confirm the activation of the two pathways by ING5 knockdown. In consistence with the antibody array, results of western blot showed that ING5 knockdown increased phosphorylation of Akt at both S473 and T308 and phosphorylation of STAT3 at Y705, which indicated activation of PI3K/Akt and STAT3 signaling pathways. Overexpression of ING5 in lung cancer A549 and H1299 cells and colorectal cancer HCT116 cells significantly reversed activation of both PI3K/Akt and STAT3 pathways. EGFR and IL-6, which are known activators of PI3K/Akt and STAT3 signaling pathways respectively, were upregulated in ING5 knockdown cells, while downregulated in ING5 overexpressing cells (Figure 2C).

### Inhibition of PI3K or STAT3 pathway reverses ING5 knockdown-induced invasiveness of lung cancer cells

**Figure 3:**
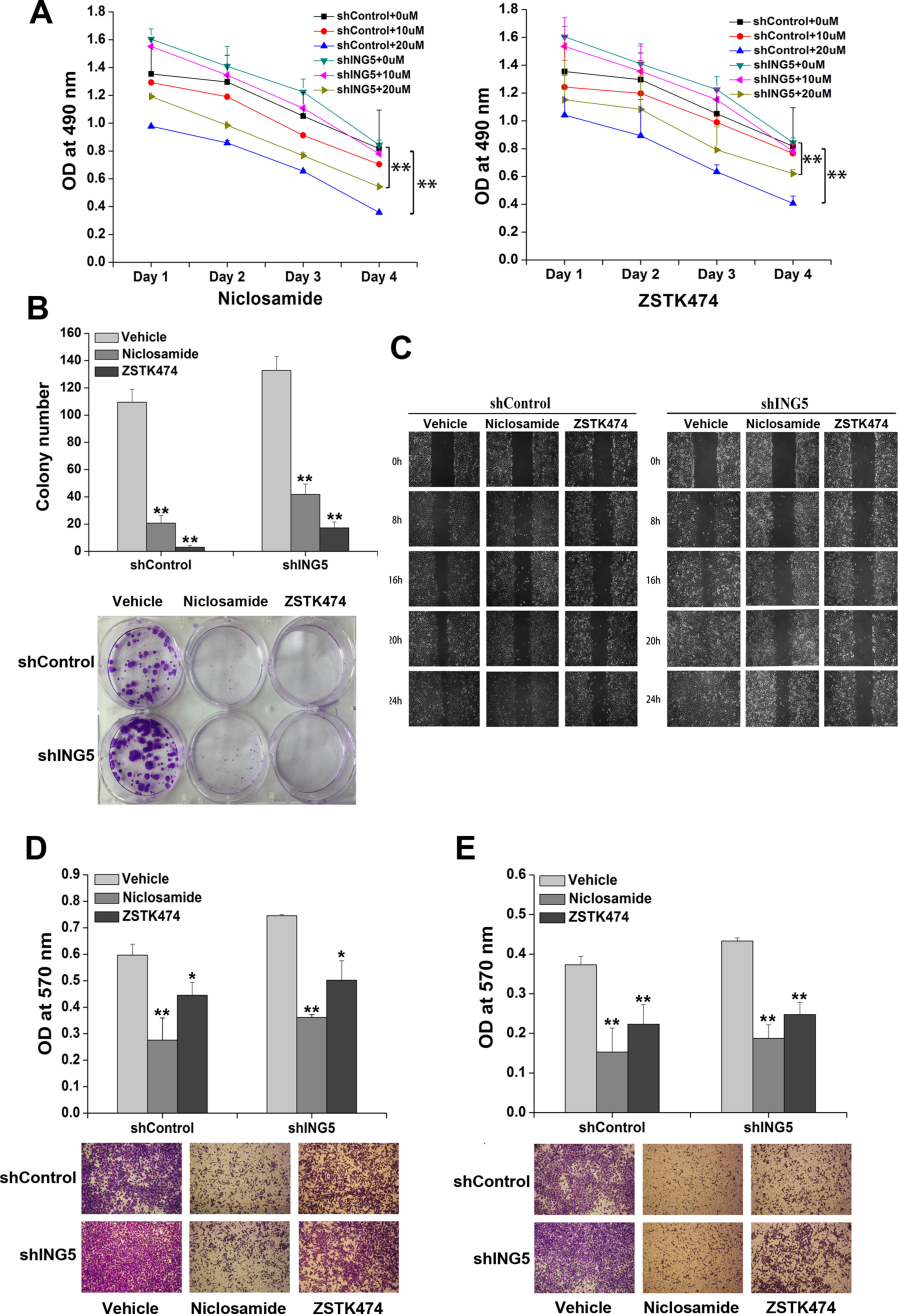
Inhibition of STAT3 or PI3K/Akt pathway reverses ING5 knockdown-promoted cancer aggressiveness (**A**) Effects of STAT3 inhibitor Niclosamide or PI3K inhibitor ZSTK474 on proliferation of shControl and shING5 A549 cells. **P < 0.01 compared to corresponding vehicle control. *P* = 0.03 and *P* = 0.02, comparing shING5 cells with shControl cells treated with 10 μM or 20 μM Niclosamide, respectively. *P* = 0.35 and *P* = 0.02, comparing shING5 cells with shControl cells treated with 10 μM and 20 μM ZSTK474, respectively. (**B**) Effects of Niclosamide and ZSTK474 on colony formation abilities of shControl and shING5 A549 cells. Representative pictures are shown. Data are shown as mean plus standard error of three independent experiments. **P < 0.01 compared to corresponding vehicle control. *P* = 0.04 and *P* = 0.001, comparing shING5 cells with shControl cells treated with 20μM Niclosamide and ZSTK474, respectively. (**C**) Wound-healing assay was performed to show the effects of Niclosamide and ZSTK474 on migration of A549 shControl and shING5 cells. A scratch wound was made on cell surface and cells were photographed at 0 h, 8 h, 16 h, 20 h and 24 h. Representative pictures are shown. (**D**) Effects of Niclosamide or ZSTK474 on transwell migration of A549 shControl and shING5 cells. The migrated cells were photographed (100 × magnification). Representative pictures are shown. Data are shown as mean plus standard error of three independent experiments. *P < 0.05 and **P < 0.01 compared to corresponding vehicle control. *P* = 0.02 and *P* = 0.03, comparing shING5 cells with shControl cells treated with 20 μM Niclosamide and ZSTK474, respectively. (**E**) Effects of Niclosamide and ZSTK474 on invasive abilities of A549 shControl and shING5 cells. The invaded cells were photographed (100 × magnification). **P < 0.01 compared to corresponding vehicle control. *P* = 0.02 and *P* = 0.02, comparing shING5 cells with shControl cells treated with 20 μM Niclosamide and ZSTK474, respectively.

ZSTK474 and Niclosamide are novel inhibitors of PI3K/AKT and STAT3 signaling pathways respectively with potent antitumor activity and low toxicity both *in vivo* and *in vitro* [23–26]. To investigate whether ING5 inhibits cancer cell invasiveness by targeting both signaling pathways, we treated A549 shControl and A549 shING5 cells with ZSTK474 and Niclosamide, respectively. The effects of ZSTK474 and Niclosamide on cell proliferation and invasion were observed. The results showed that both ZSTK474 and Niclosamide treatments significantly inhibited cell proliferation and colony formation abilities of A549 shControl and shING5 cells (Figure 3A, 3B). Furthermore, both inhibitors could suppress migration of A549 shControl and shING5 cells assessed by wound-healing assay and transwell migration assay (Figure 3C, 3D). In addition, ZSTK474 and Niclosamide also significantly prevented A549 shControl and shING5 cells from invading through Matrigel-coated polycarbonate filter in the transwell chamber (Figure 3E). These results demonstrated that both ZSTK474 and Niclosamide could reverse the invasive abilities of lung cancer cells promoted by ING5 knockdown.

### Inhibition of PI3K or STAT3 pathway prevents metastasis of ING5 knockdown lung cancer cells in mouse xenograft models

**Figure 4:**
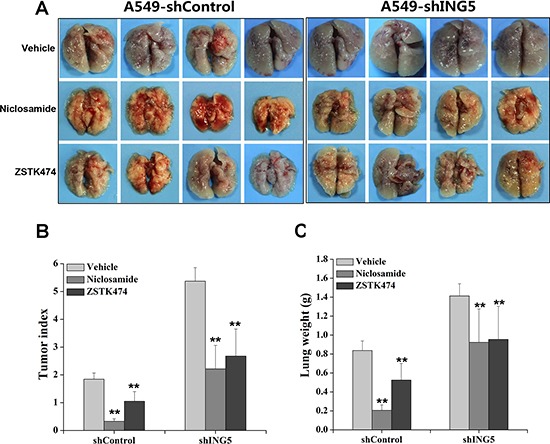
Inhibition of STAT3 or PI3K/Akt pathway impairs ING5 knockdown-promoted cancer cell metastasis *in vivo* (**A**) Mice were injected through tail vein with 5 × 10^6^ A549 shControl cells or A549 shING5 cells. At day 40 after tumor cell injection, mice were sacrificed and photographed. Gross images of lung show lung-metastasized tumors in shControl and shING5 groups of mice with or without treatments. Representative pictures are shown. (**B**) Tumor index of mice from different groups. **P < 0.01 compared to corresponding vehicle control. *P* = 0.005 and *P* = 0.0001, comparing shING5 cells with shControl cells treated with Niclosamide and ZSTK474, respectively. (**C**) Lung weight of mice from different groups. **P < 0.01 compared to corresponding vehicle control. *P* = 0.002 and *P* = 0.01, comparing shING5 cells with shControl cells treated with Niclosamide and ZSTK474, respectively.

To investigate whether PI3K/AKT and STAT3 signaling pathways were involved in ING5 knockdown-stimulated lung cancer invasiveness *in vivo*, we made intravenous mouse xenograft model by injecting A549 shControl and A549 shING5 cells through tail veins of nude mice. Mice were sacrificed at day 40 after injection and lungs were separated, weighed and inspected for tumor formation. All mice that were injected with A549 shControl and A549 shING5 cells developed multiple tumors in bilateral lungs (Figure 4A). Tumor index was calculated on the basis of a grading system described by other group [27]. The vehicle control mice injected with A549 shControl had significantly lower tumor index and lung weights compared with vehicle control mice injected with A549 shING5 cells. The mice treated with Niclosamide or ZSTK474 had significantly lower tumor index (Figure 4B) and lung weights (Figure 4C) compared with corresponding vehicle control groups.

### Inhibition of PI3K or STAT3 pathway reverses ING5 knockdown-induced EMT

**Figure 5:**
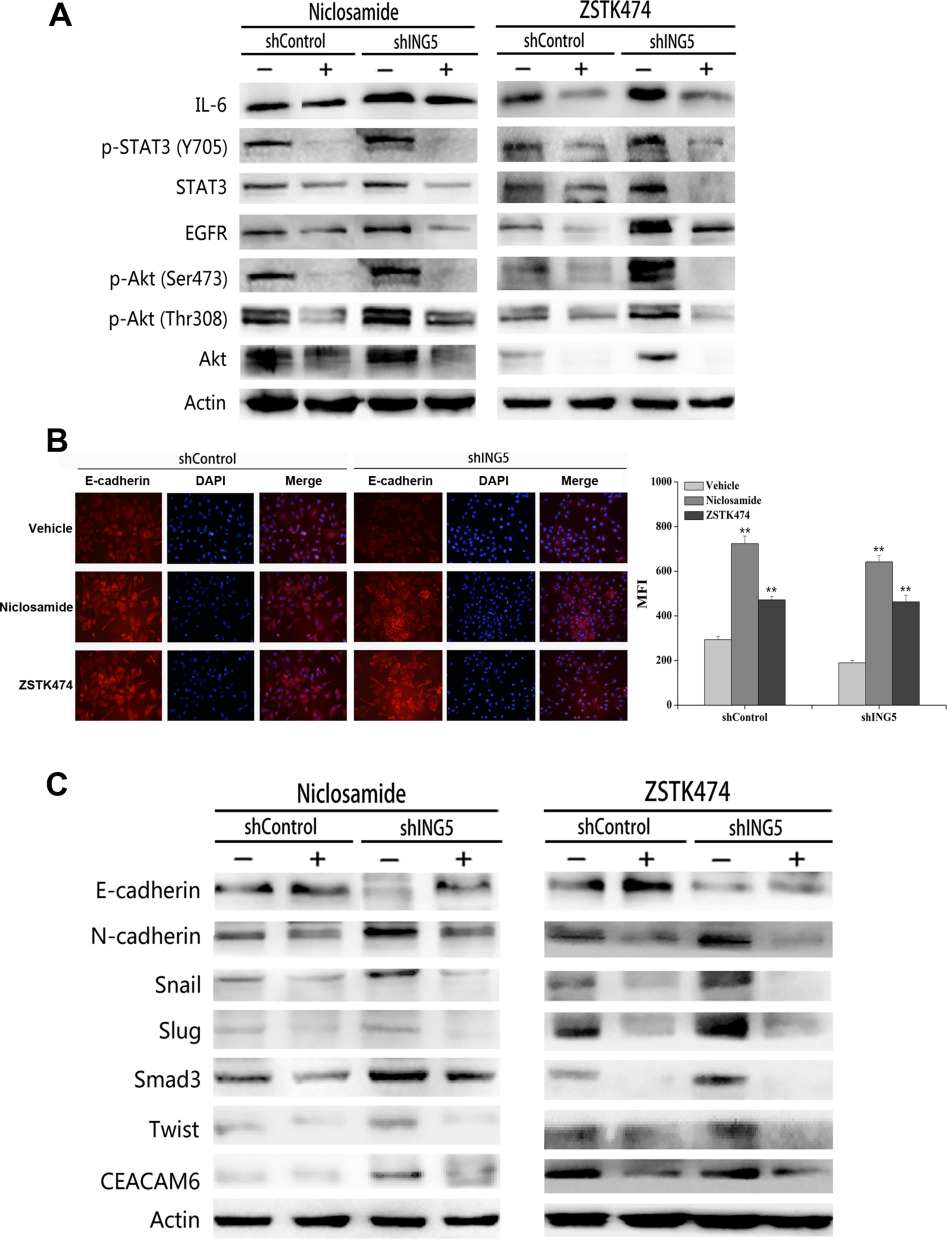
Inhibition of STAT3 or PI3K/Akt pathway reverses ING5 knockdown-induced EMT (**A**) Effects of Niclosamide or ZSTK474 on expression of proteins involved in EGFR/PI3K/Akt and IL-6/STAT3 pathways by western blot. Actin was used as an internal loading control. (**B**) Effects of Niclosamide or ZSTK474 on the expression of E-cadherin by immunofluorescence staining in shControl and shING5 A549 cells. Representative pictures are shown (200× magnification). Data are shown as mean plus standard error of at least three independent experiments. The MFI value (mean fluorescence intensity) was compared. **P < 0.01 compared to corresponding vehicle control. *P* = 0.01 and *P* = 0.04, comparing shING5 cells with shControl cells treated with Niclosamide and ZSTK474, respectively. (**C**) Effects of Niclosamide or ZSTK474 on protein expression of EMT markers and EMT-related proteins by western blot. Actin was used as an internal loading control.

To explore whether ING5 knockdown induces EMT via activation of PI3K/Akt and STAT3 pathways, we treated shControl and ING5 knockdown A549 cells with inhibitors of both pathways. ZSTK474 or Niclosamide significantly decreased phosphorylated STAT3 and Akt, as well as EGFR and IL-6 in both shControl and shING5 A549 cells (Figure 5A). Both inhibitors increased the epithelial marker E-cadherin in shControl and ING5 knockdown A549 cells by immunofluorescence staining and western blot (Figure 5B, 5C), while downregulated the mesenchymal marker N-cadherin, EMT-related transcription factors Snail, Slug, Smad3 and Twist, and EMT-inducing protein CEACAM6 by western blot (Figure 5C). These results suggest that the EGFR/PI3K/Akt and IL-6/STAT3 signaling pathways play important roles in ING5 knockdown-induced EMT and tumor aggressiveness in lung cancer cells.

## DISCUSSION

ING5 has been reported to be involved in regulation of cell cycle progression, apoptosis and differentiation [5, 7, 9], and chromatin modification as a component of HAT complexes [6] since it was first identified 14 years ago. Previously, we have shown that overexpression of ING5 significantly inhibited lung cancer cell invasiveness by preventing EMT [10]. In the current study, we further confirm that loss of ING5 promotes EMT and cancer invasion, suggesting that downregulation of the tumor suppressor gene ING5 potentiates the invasive ability of lung cancer cells by promoting EMT. Our results indicate an indispensible role of ING5 in preventing EMT and metastasis in lung cancer.

EMT is considered as one of the most critical steps in cancer metastasis [28–30], which has been proved to promote cell proliferation, survival, differentiation, cancer stem cells “stem feature” and drug resistance [31], thus playing an important role in cancer malignancy, metastasis and recurrence. Oncogenic kinase signaling pathways, including PI3K/Akt and STAT3, are often upregulated in cancer initiation and invasion. IL-6/STAT3 pathway has been confirmed to induce EMT and metastasis in many types of cancers [22, 32]. EGFR is another molecule which has been clearly shown positively associated with cancer invasion by activating downstream PI3K/Akt signaling and inducing EMT [33, 34]. There is crosstalk between the two pathways in EMT progression, as EGF treatment enhances IL-6 production [35], and elevated level of IL-6 is associated with cancer cell aggressiveness and metastasis by inducing EMT through activating STAT3 and Akt signaling [36].

In the current study, by Phospho-Kinase antibody array and western blot, we have screened and confirmed upregulated phosphorylation of Akt S473/T308 and STAT3 Y705 upon ING5 knockdown. We also have found increased EGFR and IL-6 protein level in ING5 knockdown A549 cells, which function upstream of PI3K/Akt and STAT3 signaling and activate both pathways, respectively. ING5 overexpression significantly downregulates both EGFR/PI3K/Akt and IL-6/STAT3 pathways. Treatment with PI3K inhibitor or STAT3 inhibitor prevented activation of both signals and caused a reversal of EMT and metastatic phenotypes induced by ING5 knockdown. These results clearly demonstrate that ING5 functions as a tumor suppressor by inhibiting EGFR/PI3K/Akt and IL-6/STAT3 signaling pathways.

The mechanisms by which ING5 suppresses both PI3K/Akt and STAT3 pathways need further investigation. The current data show that ING5 overexpression downregulates not only phophorylated Akt and STAT3, but also total protein level of both proteins, and knockdown of ING5 upregulates both phosphorylated and total Akt and STAT3. These results may suggest that ING5 regulates the activity of both pathways by an indirect way. Our previous study has found elevated IL-6 mRNA level in ING5 knockdown A549 cells by cDNA array, which was further confirmed by qRT-PCR with 6.63 fold higher in shING5 A549 cells than in shControl cells, while EGFR mRNA was not increased by ING5 depletion [10]. It's very interesting to find ING5 overexpression significantly decreases EGFR protein level, which is upregulated in ING5 knockdown cells. We speculate that ING5 overexpression might decrease EGFR protein stability by regulating EGFR post-translational modification, which are under further investigation.

In conclusion, our results demonstrate, for the first time that ING5 knockdown induces EMT by activating EGFR/PI3K/Akt and IL-6/STAT3 oncogenic signaling pathways, leading to increased cancer invasion and metastasis, thus proposing a promising role of ING5 in anti-metastasis therapy for lung cancer patients.

## MATERIALS AND METHODS

### Cell culture and reagents

Human lung cancer cell lines (A549, H1299) and Human colorectal cancer HCT116 cell line were purchased from the Type Culture Collection of the Chinese Academy of Sciences, Shanghai, China. These cells were grown in Dulbecco's modified Eagle's medium (DMEM, Gibco, USA) supplemented with 10% fetal bovine serum (HyClone, USA), 10 mg/ml antibiotics (penicillin and streptomycin) and 2 mmol/L L-glutamine at 37°C under 5% CO_2_ and saturated moisture. The establishment of cell lines with ING5 stable overexpression or knockdown were described previously [10]. ZSTK474 and Niclosamide were purchased from Selleck (Houston, TX, USA). These compounds were dissolved in dimethylsulfoxide (DMSO) as a solid dispersion form for cell and animal experiments.

### Proliferation assay

Cells were seeded in triplicate in 96-well culture plates at a density of 5 × 10^4^ cells/200 μL/well. Cells were added MTT solution (5 mg/mL) at different time points and continuously incubated at 37°C, 5% CO_2_ incubator for 4 hours. Supernatants were removed from these wells and 150 μL of DMSO was added to each well. After shaking for 10 minutes at room temperature, the absorption was read immediately at 490 nm with a microplate reader (iMarkTM, Bio Rad, Hercules, CA, USA). The number of viable cells remaining after the treatment was calculated using the following formula: Cell number (% control) = 100 × (absorbance of a given sample-absorbance of blank well)/(absorbance of control well-absorbance of blank well), where the blank well contained medium but no cells and the control well contained cells but no drugs. Experiments were done in triplicate and analyzed by paired *t*-test.

### Colony formation assay

Cells were seeded in 6-well culture plates at a density of 3 × 10^2^ cells/2 mL/well and incubated for 15 days when colonies were visible. Crystal violet staining was performed and the number of colonies was counted.

### Wound-healing assay

Cells were seeded in 6-well culture plates at a density of 4 × 10^5^ cells/2 mL/well. Once the cells reached 90% confluence, a wound area was carefully created by scraping the cell monolayer with a sterile 200 μL pipette tip, from one end to the other end of the well. The detached cells were removed by washing with PBS. Cells migrated to the wounded region were observed by Olympus CK-2 inverted microscope and photographed (100 × magnification) at 0 h, 8 h, 16 h, 20 h and 24 h. The experiments were performed in triplicate.

### Transwell migration and invasion assay

For the migration assay, 5 × 10^4^ cells were suspended in 200 μL serum-free medium and plated on chambers (Corning Costar, NY, USA) that were not coated with Matrigel. For the invasion assay, the upper chamber was pre-coated with Matrigel (BD Bioscience, CA, USA) according to the manufacturer's protocols before 5 × 10^4^ cells in 200 μL serum-free DMEM were added to the chamber. For both assays, 600 μL medium containing 10% FBS was added to the lower chamber as a chemo-attractant. The cells were incubated for 14 h (migration) or 24 h (invasion). Non-invasive cells in the upper chamber were removed by wiping with a cotton swab, and invasive cells were fixed with 4% formaldehyde in PBS and were stained with 1% crystal violet in 2% ethanol. Cells in the lower surface of the filter were photographed under a light microscope (100 × magnification). The inserts were washed with 33% acetic acid. Absorbance of washing buffer at 570 nm was determined for each well using a microplate reader. Cell-free inserts containing only medium had been included in duplicate throughout each experiment as OD background controls. Reported OD data represent average background-corrected values ± SD obtained from three independent experiments in duplicate.

### Western blot

Cells were lysed in lysis buffer containing 150 mM NaCl, 1% NP40, 0.5% deoxycholic acid, 0.1% SDS, 50 mM Tris ( pH 8.0), and 1:25 protease inhibitor cocktail for total protein. Protein concentrations of the lysates were determined by the Bradford protein assay system (Bio-Rad, Hercules, CA). Equal amounts of protein (30 μg protein each lane) were separated by SDS-PAGE and transferred to nitrocellulose membranes (Hybond C, Amersham, UK). Immunoblots were blocked with 5% skim milk in TBS/Tween 20 (0.05%, v/v) for 1 hour at RT. The membrane was incubated with primary antibody overnight at 4°C. Primary antibodies used include antibodies for ING5 and SMAD3 (Proteintech Group, Inc.), IL-6 (Bioworld technology, Inc.), p-AKT (Ser473, Thr308), STAT3 and p-STAT3 (Tyr705) (Cell Signaling, Inc.), AKT, E-cadherin, N-cadherin, Snail, Slug, Twist, EGFR and CEACAM6 (Abcam), and β-actin (Actin) (Sigma). The membrane was incubated with corresponding secondary antibody conjugated with horseradish peroxidase (Sigma) (1:5000) at RT for 1h. The blots were developed using an enhanced chemiluminescence western blot detection system (Amersham Bioscience, UK).

### Phospho-kinase array

To analyze the phosphorylation profiles of proteins influenced by ING5 knockdown, we did antibody array with Human Phospho-Kinase Array Kit (R&D Systems, ARY003B) with 43 kinase phosphorylation sites according to the manual. Briefly, lysates from A549 shControl and A549 shING5 cells were incubated with the array. The array was then washed to remove unbound proteins followed by incubation with a cocktail of biotinylated detection antibodies. Streptavidin-HRP and chemiluminescent detection reagents were applied to produce a signal at each capture spot corresponding to the amount of phosphorylated protein. Pixel densities on developed film were scanned and analyzed.

### Xenograft studies

Male athymic nude mice (6 weeks old) were bought from Experimental Animal Center of Fourth Military Medical University. All animal procedures were performed in accordance with protocols approved by the Animal Care and Use Committee of Fourth Military Medical University. Mice were injected with 5 × 10^6^ A549 shControl cells or A549 shING5 cells through tail vein. Treatment was initiated on the day after injection and administered daily for 10 days with ZSTK474 50 mg/kg/day orally and Niclosamide 20 mg/kg/day intraperitoneally. Mice were sacrificed at day 40 after injection and lungs were inspected for tumor formation.

### Immunofluorescence staining

Cells were trypsinized and seeded on sterile coverslips placed in 24-well culture plates at a density of 3 × 10^4^ cells/600 μL/well and treated with 20 μM ZSTK474, Niclosamide or DMSO as vehicle for 8 hours. Cells were then fixed with 4% paraformaldehyde for 15 minutes at room temperature, and permeabilized with 0.5% Triton X-100 for 10 minutes. After blocking with 5% BSA for 1 hour at room temperature, cells were incubated with the primary antibody (rabbit monoclonal antibody against E-cadherin 1:100) for overnight at 4°C. Cells were then incubated with conjugated goat anti-rabbit secondary antibody (1:1000) for 1 hour at room temperature in the dark. To ensure specificity of our results, negative controls with no primary antibody or no secondary antibody were included. For nuclear counterstaining, cells were incubated with DAPI (1:50) for 5 minutes. Coverslips were then mounted with Fluoromount-G. Cells were visualized using Zeiss LSM510 Meta confocal microscope (Carl Zeiss Microscopy GmbH, Germany). Images were acquired at 200 × total magnification using Zeiss Zen 2009 software.

### Statistical analysis

Two groups of data were analyzed by *t*-test. All of the statistical tests were 2-sided. All data were analyzed with SPSS 17.0 software program. *P* < 0.05 was regarded as statistically significant.

## SUPPLEMENTARY MATERIALS FIGURES AND TABLES


