# Supplementary material for: ING5 knockdown enhances migration and invasion of lung cancer cells by inducing EMT via EGFR/PI3K/Akt and IL-6/STAT3 signaling pathways
Source: Oncotarget. 2017 Apr 21;8(33):54265–76. doi: 10.18632/oncotarget.17346 (PMC5589578; doi:10.18632/oncotarget.17346)
Supplement: Supplementary file 1 [file oncotarget-08-54265-s001.pdf]

# ING5 knockdown enhances migration and invasion of lung cancer cells by inducing EMT via EGFR/PI3K/Akt and IL-6/STAT3 signaling pathways

## Supplementary Materials

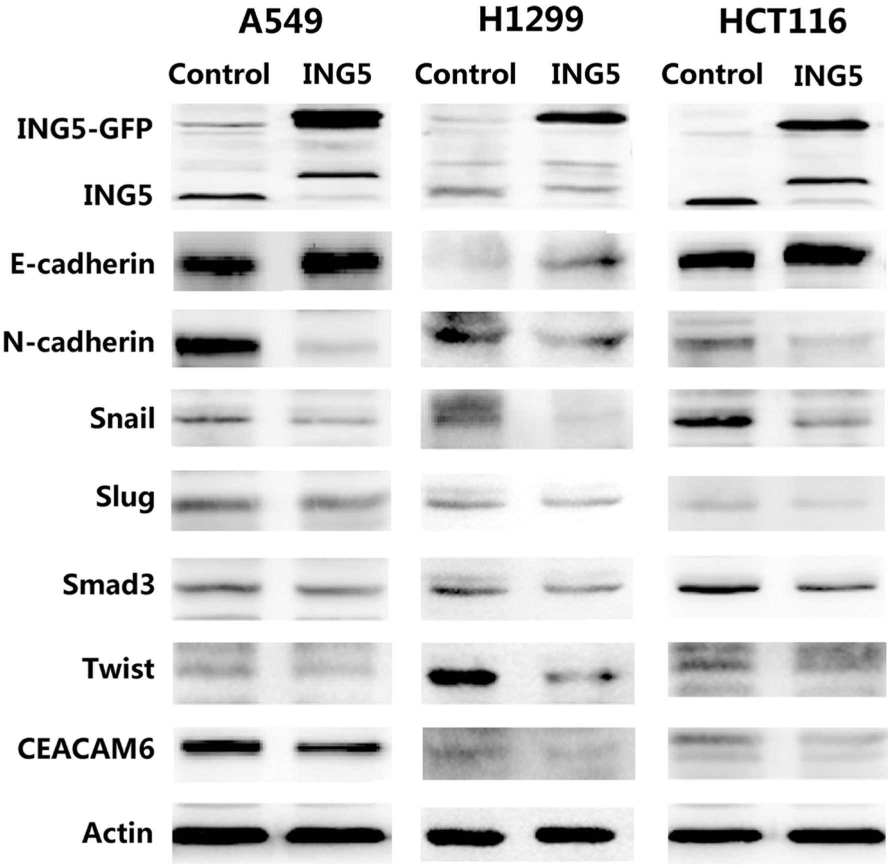


**Supplementary Figure 1: Effects of ING5 overexpression on protein level of EMT markers and related proteins in A549, H1299 and HCT116 cells by western blot.** Actin was used as an internal loading control.

## Human Phospho-Kinase Array Coordinates

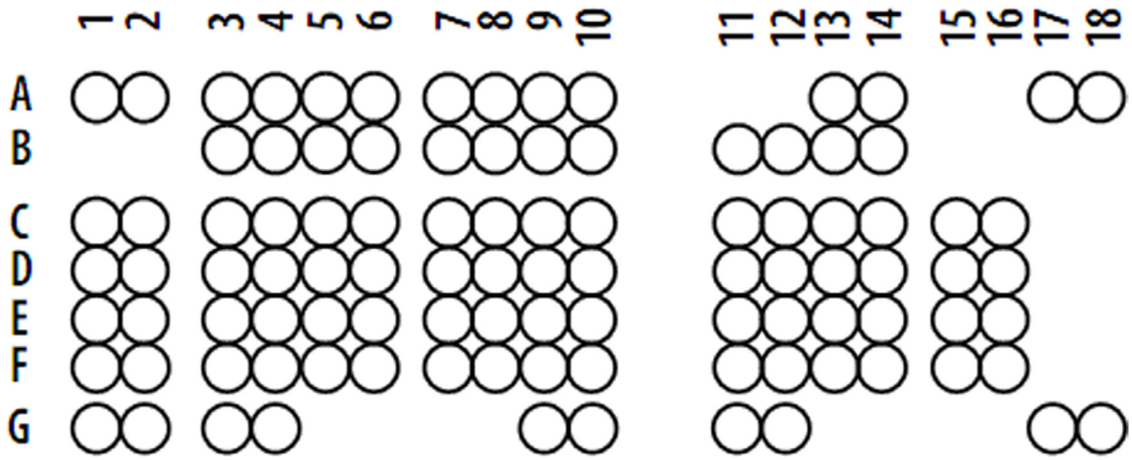


**Supplementary Figure 2: Human Phospho-Kinase Array coordinates.**

## Supplemental Table 1: Human phosphor-kinase array coordinates and ratio of shING5/shControl

| Coordinate | Target | Phosphorylation site | shING5/shControl |
|---|---|---|---|
| A1, A2 | Reference Spot | | 1 |
| A3, A4 | P38α | T180/Y182 | 0.89 |
| A5,A6 | ERK1/2 | T202/Y204, T185/Y187 | 1.66 |
| A7, A8 | JNK1/2/3 | T183/Y185, T221/Y223 | 1.09 |
| A9, A10 | GSK-3α/β | S21/S9 | 1.01 |
| A13, A14 | p53 | S392 | 2.12 |
| A17, A18 | Reference Spot | | 1 |
| B3, B4 | EGFR | Y1086 | 1.19 |
| B5, B6 | MSK1/2 | S376/S360 | 1.25 |
| B7, B8 | AMPKα1 | T183 | 0.96 |
| B9, B10 | Akt1/2/3 | S473 | 1.65 |
| B11, B12 | Akt1/2/3 | T308 | 1.83 |
| B13, B14 | p53 | S46 | 1.96 |
| C1, C2 | TOR | S2448 | 1.54 |
| C3, C4 | CREB | S133 | 0.80 |
| C5, C6 | HSP27 | S78/S82 | 1.06 |
| C7, C8 | AMPKα2 | T172 | 1.62 |
| C9, C10 | β-Catenin | *** | 1.23 |
| C11, C12 | p70 S6 Kinase | T389 | 1.13 |
| C13, C14 | p53 | S15 | 1.67 |
| C15, C16 | c-Jun | S63 | 1.18 |
| D1, D2 | Src | Y419 | 1.17 |
| D3, D4 | Lyn | Y397 | 1.32 |
| D5, D6 | Lck | Y394 | 1.08 |
| D7, D8 | STAT2 | Y689 | 2.36 |
| D9, D10 | STAT5a | Y694 | 1.59 |
| D11, D12 | p70 S6 Kinase | T421/S424 | 1.63 |
| D13, D14 | RSK1/2/3 | S380/S386/S377 | 1.35 |
| D15, D16 | eNOS | S1177 | 1.30 |
| E1, E2 | Fyn | Y420 | 1.21 |
| E3, E4 | Yes | Y426 | 1.08 |
| E5, E6 | Fgr | Y412 | 1.10 |
| E7, E8 | STAT6 | Y641 | 1.62 |
| E9, E10 | STAT5b | Y699 | 1.72 |
| E11, E12 | STAT3 | Y705 | 1.79 |
| E13, E14 | p27 | T198 | 1.16 |
| E15, E16 | PLC-γ1 | Y783 | 1.34 |
| F1, F2 | Hck | Y411 | 1.28 |
| F3, F4 | Chk-2 | T68 | 1.17 |
| F5, F6 | FAK | Y397 | 1.15 |
| F7, F8 | PDGF Rβ | Y751 | 1.16 |
| F9, F10 | STAT5a/b | Y694/Y699 | 1.98 |
| F11, F12 | STAT3 | S727 | 1.62 |
| F13, F14 | WNK1 | T60 | 1.37 |
| F15, F16 | PYK2 | Y402 | 1.15 |
| G1, G2 | Reference Spot | | |
| G3, G4 | PRAS40 | T246 | 2.58 |
| G9, G10 | PBS (Negative Control) | | |
| G11, G12 | HSP60 | *** | 1.14 |
| G17, G18 | PBS (Negative Control) | | |

***Antibody against total protein was used.
